# Transcription Factor Id1 Plays an Essential Role in Th9 Cell Differentiation by Inhibiting Tcf3 and Tcf4

**DOI:** 10.1002/advs.202305527

**Published:** 2023-10-22

**Authors:** Woo Ho Lee, Kyung Jin Hong, Hua‐Bing Li, Gap Ryol Lee

**Affiliations:** ^1^ Department of Life Science Sogang University 35 Baekbeom‐ro Mapo‐gu Seoul 04107 South Korea; ^2^ Shanghai Institute of Immunology, State Key Laboratory of Oncogenes and Related Genes Shanghai Jiao Tong University School of Medicine 280 Chongqing South Rd, Building #5‐602 Shanghai 200025 China

**Keywords:** Id1, Th9 cell, differentiation, transcriptional regulation, Tcf3, Tcf4

## Abstract

T helper type 9 (Th9) cells play important roles in immune responses by producing interleukin‐9 (IL‐9). Several transcription factors are responsible for Th9 cell differentiation; however, transcriptional regulation of Th9 cells is not fully understood. Here, it is shown that Id1 is an essential transcriptional regulator of Th9 cell differentiation. Id1 is induced by IL‐4 and TGF‐β. *Id1‐*deficient naïve CD4 T cells fail to differentiate into Th9 cells, and overexpression of Id1 induce expression of IL‐9. Mass spectrometry analysis reveals that Id1 interacts with Tcf3 and Tcf4 in Th9 cells. In addition, RNA‐sequencing, chromatin immunoprecipitation, and transient reporter assay reveal that Tcf3 and Tcf4 bind to the promoter region of the *Il9* gene to suppress its expression, and that Id1 inhibits their function, leading to Th9 differentiation. Finally, *Id1‐*deficient Th9 cells ameliorate airway inflammation in an animal model of asthma. Thus, Id1 is a transcription factor that plays an essential role in Th9 cell differentiation by inhibiting Tcf3 and Tcf4.

## Graphical abstract

This study discovers that Id1 is a key transcription factor for Th9 differentiation. Id1 is induced specifically by IL‐4 and TGF‐β. *Id1‐*deficient CD4 T cells fail to differentiate into Th9 cells, and Id1‐overexpression induce IL‐9. Id1 bounds and inhibits Tcf3 and Tcf4, which bind to the *Il9* gene and suppress its expression. *Id1‐*deficient Th9 cells ameliorate airway inflammation in vivo.

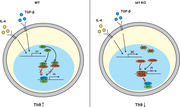

## 1 Introduction

T helper cells play an important role in protecting the body against various pathogens by coordinating immune responses. T helper cells comprise several subsets that show distinct cytokine profiles.^[^ ^1^ ^]^ These subsets include T helper type I (Th1), Th2, Th9, and Th17 cells. Th9 cells are a major producer of IL‐9 (although other subsets produce small amounts) and mediate immune responses against helminths.^[^ ^2^ ^]^ In addition, Th9 cells cause allergic reactions^[^ ^3^ ^]^ and induce antitumor immune responses.^[^ ^4^ ^]^ The IL‐4 and TGF‐β signaling pathways are important for differentiation of naïve CD4 T cells into Th9 cells.^[^ ^5^ ^]^ IL‐4 induces expression of STAT6, GATA3, and IRF4,^[^ ^6^ ^]^ all of which are essential for Th9 cell differentiation. However, IL‐4 alone induces differentiation of naïve CD4 T into Th2 cells. In the presence of IL‐4, TGF‐β induces PU.1, which inhibits Th2 pathways and promotes differentiation of Th9 cells.^[^ ^6^, ^7^ ^]^ However, TGF‐β alone induces regulatory T (Treg) cells. Thus, the combination of IL‐4 and TGF‐β induces naïve CD4 T cells to differentiate into Th9 cells, rather than into Th2 and Treg cells.

Several transcription factors are involved in Th9 cell differentiation. PU.1, induced by TGF‐β, is essential for Th9 cell differentiation.^[^ ^6^, ^7^, ^8^ ^]^ One of its role is to recruit histone acetyltransferase to the *Il9* promoter.^[^ ^9^ ^]^ IRF4 is also required for Th9 cell differentiation,^[^ ^3a^ ^]^ as well as for Th2 and Th17 cell differentiation. IRF4 binds directly to the *Il9* promoter to increase its transcription.^[^ ^3a^ ^]^ BATF is also important for Th9 differentiation,^[^ ^10^ ^]^ as well as for Th17 differentiation. Several other transcription factors, including Notch,^[^ ^7b^ ^]^ NF‐κB,^[^ ^11^ ^]^ NFAT,^[^ ^12^ ^]^ Bcl6,^[^ ^13^ ^]^ Foxo1,^[^ ^14^ ^]^ Id3,^[^ ^15^ ^]^ HIF1α,^[^ ^16^ ^]^ Etv5,^[^ ^17^ ^]^ BATF3,^[^ ^18^ ^]^ and DBP ^[^ ^19^ ^]^ regulate Th9 cell differentiation (reviewed in ref. [20]). Although several transcription factors drive Th9 cell differentiation, transcriptional regulation of Th9 cells remains poorly understood. In particular, it is not clear whether these transcription factors act as lineage‐determining transcription factors in a manner similar to T‐bet for Th1 cells, GATA3 for Th2 cells, RORγt for Th17 cells, and Foxp3 for Treg cells.^[^ ^2b^ ^]^

The inhibitor of DNA‐binding (Id) proteins Id1–Id4 are helix–loop–helix (HLH) transcription factors.^[^ ^21^ ^]^ Id proteins bind to basic HLH (bHLH) proteins and regulate their transcriptional activity by preventing them from binding to DNA.^[^ ^21^ ^]^ However, Id proteins cannot regulate transcription of their target genes by themselves because they lack a DNA‐binding domain. The primary binding partners of Id proteins are Tcf3 (E2A), Tcf4 (E2‐2), and Tcf12 (HEB), all of which are members of the E‐protein family.^[^ ^22^, ^23^ ^]^ These are bHLH proteins that form heterodimers or homodimers with each other and bind to DNA to regulate transcription.^[^ ^23^ ^]^ E‐proteins play roles in development of B and T cells.^[^ ^24^ ^]^ As regulators of E‐proteins, Id proteins are also involved in development of B and T cells.^[^ ^25^ ^]^ In addition, Id proteins regulate other cellular processes, including cell growth and differentiation, development, migration, stemness, and tumorigenesis.^[^ ^21^ ^]^

In our previous study, we examined differentially expressed genes in Th9 cells by comparing gene expression in Th2, Th9, and Treg cells, all of which are regulated by IL‐4 and/or TGF‐β.^[^ ^26^ ^]^ *Id1* mRNA was expressed specifically in Th9 cells. In the present study, we examined the roles of Id1 in Th9 cell differentiation. Unlike Th1, 2, and 17 cells, *Id1*‐deficient naïve CD4 T cells were unable to differentiate into Th9 cells. We also found that expression of IL‐9 was dependent on *Id1* levels. Tcf3 and Tcf4 inhibited an expression of IL‐9 by Th9 cells by binding to the *Il9* promoter region. In addition, expression of *Id1, Tcf3*, and *Tcf4* was upregulated by IL‐4 and TGF‐β. Lastly, *Id1*‐deficient CD4 T cells ameliorated airway inflammation in an animal model of asthma. Collectively, these data suggest that Id1 acts as an essential positive regulator of Th9 cell differentiation by inhibiting the transcriptional activity of Tcf3 and Tcf4.

## 2 Results

### 2.1 Id1 is an Essential Positive Regulator of Th9 Cell Differentiation

**Figure 1:**
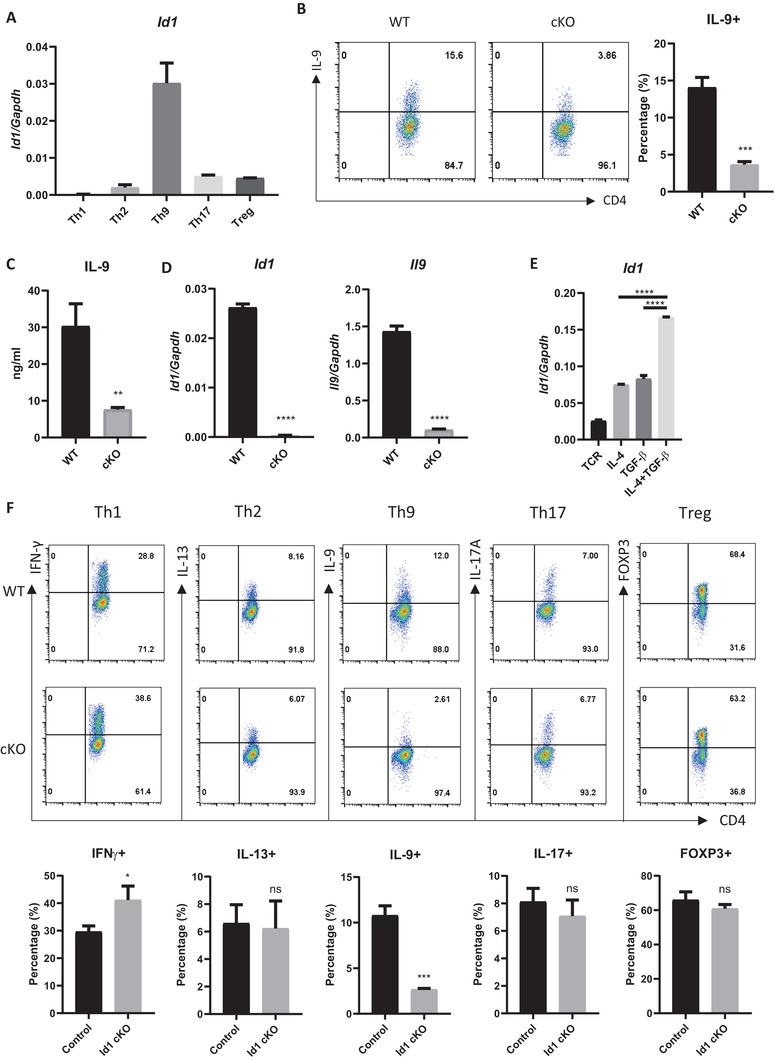
*Id1* deficiency inhibits differentiation of Th9 cells. A) Naïve CD4 T cells were isolated from the spleen of WT mice and cultured for 3 days under subset‐polarizing conditions. Expression of *Id1* mRNA was measured by RT‐qPCR. B) WT and *Id1*‐deficient naïve CD4 T cells were isolated from the spleen and cultured for 3 days under Th9‐polarizing conditions. IL‐9 was measured by flow cytometry. C) The amount of IL‐9 protein was measured by ELISA. D) *Id1* and *Il9* mRNA levels were measured by RT‐qPCR. E) Naïve CD4 T cells were isolated from the spleen and stimulated for 24 h with anti‐CD3 and anti‐CD28 antibodies in the presence of the indicated cytokines. *Id1* mRNA levels were measured by RT‐qPCR. F) Naïve CD4 T cells were isolated from the spleen of WT or *Id1* cKO mice and cultured for 3 days under subset‐polarizing conditions. Subset‐specific markers were measured by flow cytometry. B,F) Flow cytometry and A,D,E) RT‐qPCR data are representative of three independent experiments. C) ELISA data were pooled from three independent experiments. The data in the bar graph next to the flow cytometry data were pooled from three independent experiments. The error bars represent the standard deviation. B,C,D,F) *p*‐values were determined by Student's t test. E) *p*‐values were analyzed by one‐way ANOVA/Tukey's test. ns: not significant, ^*^ *p* < 0.05, ^***^ *p* < 0.001, ^****^ *p* < 0.0001.

In a previous study, to search for transcription factors that are subset‐specific, we performed differentially expressed gene analysis of Th2, Th9, and Treg cells.^[^ ^26^ ^]^ As a result, we found that Id1 is expressed specifically in Th9 cells. To confirm this, we examined *Id1* mRNA levels in various T helper subsets. *Id1* mRNA levels in Th9 cells were much higher than in other subsets (**Figure** **1**A). Next, we investigated the role of Id1 in Th9 cell differentiation using CD4 T cell‐specific *Id1*‐deficient (*Id1^fl/fl^‐Cd4^cre^*, herein named *Id1* cKO) mice. When naïve CD4 T cells from WT and *Id1* cKO mice were stimulated under Th9‐inducing conditions, *Id1*‐deficient cells expressed much smaller amounts of IL‐9 than WT cells at both the protein (Figure 1B,C) and RNA (Figure 1D) levels. We also used an independent line of mice, whole *Id1‐deficient* (whole *Id1* KO) mice, to confirm the result. Consistent with *Id1* cKO cells, whole *Id1* KO naïve CD4 T cells were unable to differentiate into Th9 cells (Figure S1A,B, Supporting Information). Next, we asked how Id1 is regulated in Th9 cells. Expression of *Id1* mRNA increased after a single treatment with IL‐4 or TGF‐β upon TCR stimulation. These two cytokines acted synergistically to increase an expression of *Id1* to levels higher than those observed after treatment with either cytokine alone (Figure 1E). Among various single or combination cytokine treatments including IL‐12, IL‐4, IL‐4+TGF‐β, IL‐6+TGF‐β, and TGF‐β, the expression of *Id1* was the highest when treated with IL‐4+TGF‐β (Figure S1C, Supporting Information). Next, to investigate the role of Id1 in differentiation of other subsets, we induced WT or *Id1* cKO naïve CD4 T cells to differentiate into Th1, 2, 9, 17, or Treg cells and then measured expression of subset‐specific markers. Interestingly, *Id1* deficiency inhibited specifically differentiation of Th9 cells (Figure 1F; Figure S1D, Supporting Information). Notably, *Id1* deficiency also enhanced differentiation of Th1 cells (Figure 1F; Figure S1D, Supporting Information). We verified the proper differentiation of all subsets through RT‐qPCR analysis (Figure S1E, Supporting Information). In addition, when cells in each subset were subjected to overexpress Id1, it resulted in enhanced differentiation specifically in Th2 and Th9 cells, while cells in other subsets remained unaffected. (Figure S1F,G, Supporting Information). Taken together, these data suggest that Id1 is an essential positive regulator of Th9 cell differentiation.

### 2.2 Differentiation of Th9 Cells is Dependent on Id1 Level and Both Id1 and Id3 are Positive Regulators of Th9 Differentiation

**Figure 2:**
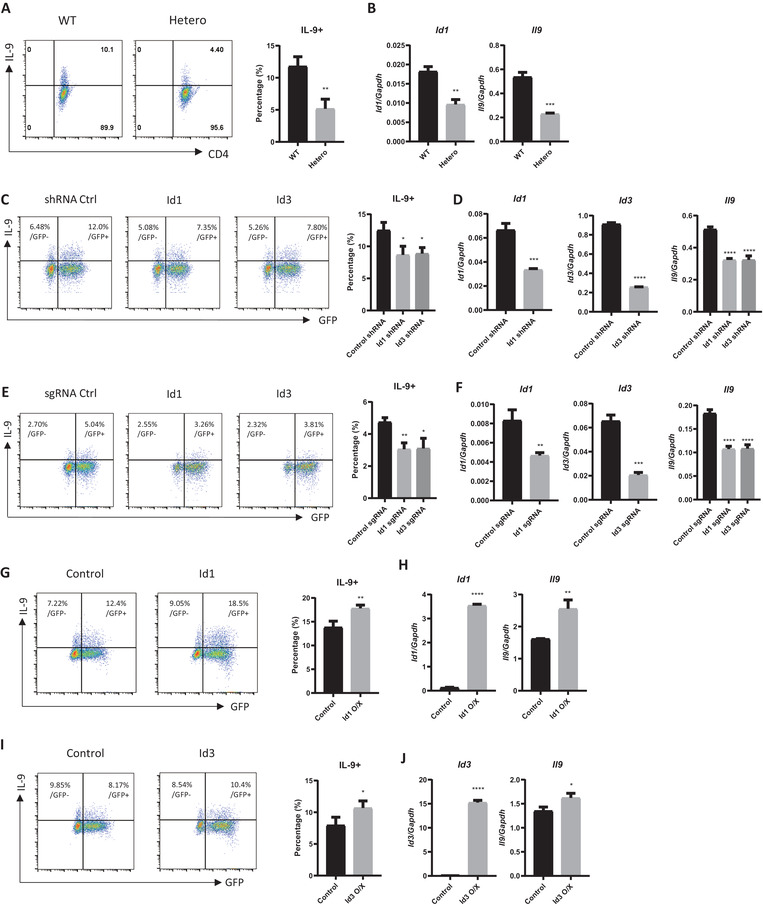
Th9 cell differentiation is dependent on the level of *Id1* expression. A,B) Naïve CD4 T cells were isolated from the spleen of *Id1^fl/fl^* or *Id1^fl/+^‐CD4^Cre^* mice and cultured for 3 days under Th9‐polarizing conditions. A) IL‐9 was measured by flow cytometry. B) *Id1* and *Il9* mRNA levels were measured by RT‐qPCR. C) Naïve CD4 T cells were isolated from the spleen of WT mice and cultured for 1 day under Th0 conditions prior to transduction with control, *Id1*, or *Id3* shRNA vectors. Cells were cultured under Th9‐polarizing conditions for 2 days. D) Expression of each gene was measured by RT‐qPCR. E) Naïve CD4 T cells were isolated from Cas9 mice and cultured for 1 day under Th0 conditions prior to transduction with control, *Id1*, or *Id3* sgRNA vectors. Cells were then cultured under Th9‐polarizing conditions for 2 days. F) Expression of each gene was measured by RT‐qPCR. G–J) Cells were cultured as described in C and transduced with control, *Id1*, or *Id3* expressing vectors. Expression of *Id1* and *Il9* was measured by RT‐qPCR. A,C,E,G,I) Flow cytometry and B,D,F,H,J) RT‐qPCR data are representative of three independent experiments. The data in the bar graph next to the flow cytometry data were pooled from three independent experiments. The error bars represent the standard deviation. A–D,G–J) *p*‐values were determined by Student's *t* test. C–F) *p*‐values were analyzed by one‐way ANOVA/Tukey's test. ns: not significant, ^*^ *p* < 0.05, ^**^ *p* < 0.01, ***p < 0.001, ^****^ *p* < 0.0001.

Next, to investigate the dosage effect of *Id1* on Th9 cell differentiation, we crossed *Id1^fl/fl^* mice with *Cd4^cre^* mice to generate *Id1^+/fl^Cd4^cre^* (*Id1* hetero) mice. Interestingly, naïve CD4 T cells from *Id1* hetero mice showed partially reduced differentiation into Th9 cells when compared with those from WT mice (**Figure** **2**A,B). To confirm that IL‐9 expression is affected by Id1 expression, we created *Id1* knockdown (KD) cells using small hairpin RNA (shRNA) or gene‐editing (GE) cells using single guide RNA (sgRNA). KD results in a partial reduction in expression of a target protein. For the sgRNA‐mediated GE, we used CRISPR‐associated protein 9 (Cas9) knockin (KI) mice that express Cas9 endonuclease. GE results from introduction of sgRNA into Cas9 KI cells. Since the probability of GE at both alleles is rare, it is expected that GE occurs partially, resulting in a status similar to heterozygosity. Both Id1 KD and GE resulted in partial reduction of Id1 expression (Figure 2C–F). Interestingly, IL‐9 expression in these cells was partially reduced, suggesting a gene dosage effect (Figure 2C–F). A previous study by Nakatsukasa et al. reported that Id3 negatively regulates Th9 cell differentiation.^[^ ^15^ ^]^ Therefore, we asked whether Id3 plays a negative role in Th9 cells. Surprisingly, and contrary to the previous report,^[^ ^15^ ^]^ inhibition of Id3 had a negative effect on Th9 cell differentiation, similar to Id1 (Figure 2C–F). Retroviral vector‐mediated overexpression of either Id1 or Id3 augmented differentiation of Th9 cells (Figure 2G–J). Overexpression of Id1 enhanced Th9 cell differentiation to a greater extent than overexpression of Id3 (Figure 2G–J). Taken together, these data suggest that Id1 and Id3 are positive regulators of Th9 cell differentiation.

### 2.3 Tcf3 and Tcf4, Binding Partners of Id1, Act as Negative Regulators of Th9 Cell Differentiation

**Figure 3:**
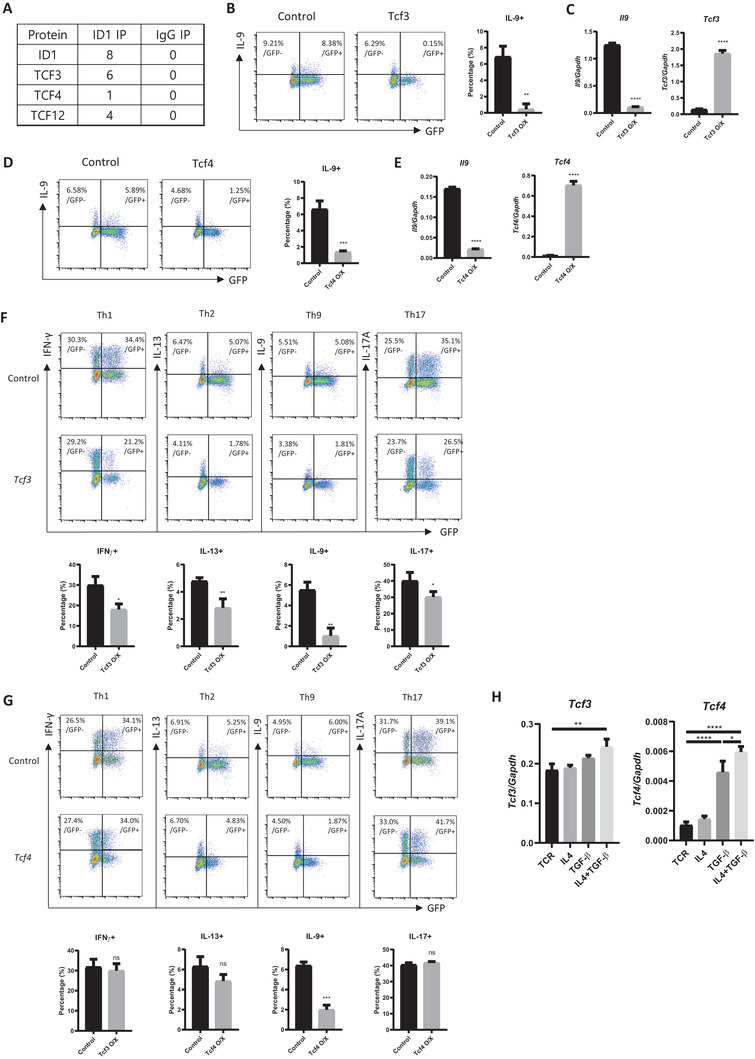
Tcf3 and Tcf4 act as negative regulators of Th9 cell differentiation. A) Id1 binding partners in Th9 cells were analyzed by mass spectrometry. The numbers indicate the number of peptides detected in the assay. B–E) Naïve CD4 T cells were transduced with *Tcf3‐* or *Tcf4‐*expressing vectors and cultured under Th9‐polarizing conditions. Next, mRNA levels were measured by RT‐qPCR. F,G) Naïve CD4 T cells were transduced with *Tcf3‐* or *Tcf4‐*expressing vectors and cultured under subset‐polarizing conditions. H) Naïve CD4 T cells were stimulated for 24 h with anti‐CD3 and anti‐CD28 antibodies in the presence of the indicated cytokines. mRNA levels were measured by RT‐qPCR. Flow cytometry and RT‐qPCR data are representative of three independent experiments. B,D,F,G) Flow cytometry and C,E,H) RT‐qPCR data are representative of three independent experiments. The data in the bar graph next to the flow cytometry data were pooled from three independent experiments. The error bars represent the standard deviation. *p*‐values were determined using Student's *t*‐test. ns: not significant, ^*^ *p* < 0.05, ^**^ *p* < 0.01, ^***^ *p* < 0.001, ^****^ *p* < 0.0001.

As mentioned above, Id1 does not have a DNA‐binding domain. Thus, we hypothesized that the Id1‐binding partners act as negative regulators of Th9 cells. To search for Id1 partners, we performed an immunoprecipitation experiment with an anti‐Id1 antibody and analyzed the coimmunoprecipitated (Co‐IPed) proteins by mass spectrometry. As a result, we found that Tcf3, Tcf4, and Tcf12, all of which are transcription factors containing a HLH domain, bind to Id1 in Th9 cells (**Figure** **3**A; Data S1, Supporting Information). When Tcf3 or Tcf4 was overexpressed in differentiating Th9 cells, IL‐9 expression fell markedly (Figure 3B–E). However, Tcf12‐overexpressing cells died (Figure S2A, Supporting Information), while Tcf3 and Tcf4 overexpressing cells did not (Figure S2B, Supporting Information). To investigate whether Tcf3 and Tcf4 affect differentiation of other subsets, we overexpressed Tcf3 or Tcf4 in activated naïve CD4 T cells cultured under Th1, Th2, Th9, and Th17 differentiation conditions. Interestingly, overexpression of Tcf3 or Tcf4 markedly reduced Th9 differentiation (Figure 3F,G). Overexpression of Tcf3 also reduced Th1, Th2, and Th17 differentiation, although less markedly than Th9 differentiation, however, overexpression of Tcf4 did not affect Th1, Th2, and Th17 differentiation (Figure 3F,G). Next, to determine whether expression of *Tcf3* and *Tcf4* in Th9 cells is regulated by cytokines, we treated TCR‐stimulated naïve CD4 T cells with IL‐4 and/or TGF‐β. Interestingly, like *Id1, Tcf3* and *Tcf4* were upregulated by TGF‐β or IL‐4 plus TGF‐β, the latter having a greater effect (Figure 3H). Taken together, these data suggest that Tcf3 and Tcf4 inhibit differentiation of Th9 cells; moreover, Tcf4 acts as a more specific negative regulator of Th9 cells than Tcf3.

### 2.4 Tcf3 and Tcf4 Inhibit Il9 Promoter Activity by Blocking Positive Transcription Factors

**Figure 4:**
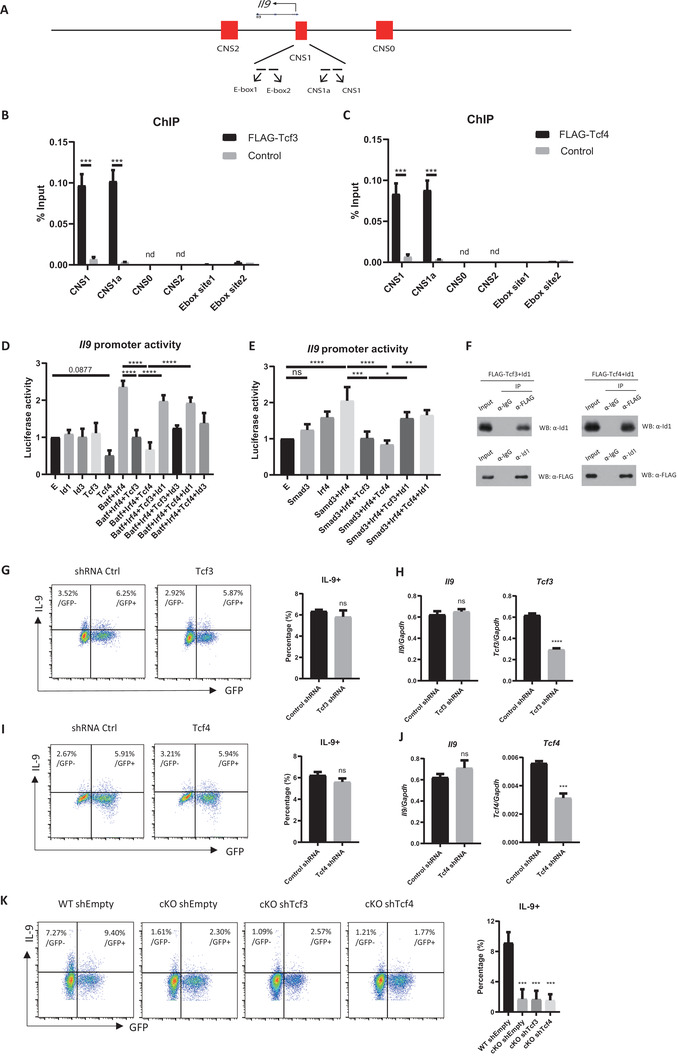
Both Tcf3 and Tcf4 are important for blockade of Th9 cell differentiation. A) Schematic diagram of the *Il9* locus. B,C) Binding of FLAG‐Tcf3 or FLAG‐Tcf4 to various regions of the *Il9* gene was measured in a chromatin immunoprecipitation (ChIP) assay of *Flag‐Tcf3‐* or *Flag‐Tcf3‐*overexpressing Th9 cells. The following regions were examined: CNS1(−375 to −270), CNS1a (−38 to −125), CNS0 (−6287 to −6093), CNS2 (+4888 to +4983), Ebox site1 (+14 to +129), and Ebox site 2 (+37 to +136). nd: not detected. (D‐E) *Il9* promoter activity was measured in EL4 cells transduced with various factors and then stimulated with PMA and ionomycin for 4 h. F) HEK293T cells were transfected with pCMV‐*Flag‐Tcf3* or pCMV‐*Flag‐Tcf4*. Cell lysates were immunoprecipitated with an anti‐Id1 or an anti‐FLAG antibody, and protein signals were measured by immunoblotting with an anti‐Id1 or an anti‐FLAG antibody. ChIP and Co‐IP data are representative of three independent experiments. G–J) Naïve CD4 T cells were transduced with the shRNA‐*Tcf3* or shRNA‐*Tcf4* vector and cultured under Th9‐polarizing conditions. mRNA levels were measured by RT‐qPCR. K) Naïve CD4 T cells were isolated from WT or *Id1* cKO mice and transduced with control, *Tcf3*‐shRNA, or *Tcf4‐* shRNA vectors prior to culture under Th9‐polarizing conditions. G,I) Flow cytometry and B,C,H,J) RT‐qPCR, F) Immunoblot data are representative of three independent experiments. D,E) Transient reporter assay data were pooled from three independent experiments. The data in the bar graph next to the flow cytometry data were pooled from three independent experiments. The error bars represent the standard deviation. B,C,G–J) *p*‐values were determined by Student's *t*‐test. D,E,K) *p*‐values were analyzed by one‐way ANOVA/Tukey's test. ns: not significant, ^***^ *p* < 0.001, ^****^ *p* < 0.0001.

To further analyze Id1‐ and Tcf3/Tcf4‐mediated expression of *Il9*, we performed a chromatin immunoprecipitation (ChIP) assay using a vector expressing FLAG‐Tcf3 or FLAG‐Tcf4. The *Il9* locus contains several regulatory regions including CNS0, CNS1, and CNS2^[^ ^27^ ^]^ (**Figure** **4**A). Tcf3 and Tcf4 bound to CNS1 and CNS1a of the *Il9* locus (Figure 4B,C). We also found that neither Tcf3 nor Tcf4 bound to the Ebox sites previously reported as binding sites for E‐proteins^[^ ^15^ ^]^ (Figure 4B,C). Since CNS1 contains the *Il9* promoter region, to which various transcription factors bind,^[^ ^27^ ^]^ we examined whether Tcf3 and Tcf4 inhibit *Il9* promoter activity by blocking transcription factor activity. For this, we used the *Il9* promoter (from −1156 to +17) used in our previous study,^[^ ^18^ ^]^ as well as the BATF and IRF4 transcription factors, which bind to the *Il9* promoter and induce its expression.^[^ ^10^ ^]^ Surprisingly, Tcf3 and Tcf4 completely abolished BATF‐IRF4‐mediated enhancement of *Il9* promoter activity (Figure 4D). However, Tcf12 did not inhibit *Il9* promoter activity (Figure S3A, Supporting Information). Tcf4 alone decreased *Il9* promoter activity. Importantly, Id1 prevented Tcf3 and Tcf4 from interfering with BATF‐IRF4, resulting in restoration of *Il9* promoter activity (Figure 4D). Id3 had only weak activity in this respect (Figure 4D). A Co‐IP experiment revealed that Id1 bound to Tcf3 and Tcf4 (Figure 4E). We also examined the impact of Tcf3 and Tcf4 on the activity of PU.1 and Smad3, both of which regulate *Il9* promoter activity.^[^ ^20a^ ^]^ Interestingly, Tcf3 and Tcf4 completely suppressed the enhancement of *Il9* promoter activity by the Smad3‐IRF4 complex (Figure 4E) but had no effect on the PU.1‐IRF4 complex (Figure S3B, Supporting Information). However, Id1 counteracted the interference of Tcf3 and Tcf4 with Smad3 and IRF4, leading to the restoration of *Il9* promoter activity (Figure 4E). The differential effects of Tcf3 and Tcf4 on these transcription factors might be attributed to their differing mode of action. Specifically, PU.1 directly binds to histone acetyltransferases Gcn5 to facilitate chromatin remodeling,^[^ ^9^ ^]^ thereby enabling transcription factors to bind to the *Il9* promoters. Conversely, Smad3 binds directly to the BATF+IRF4 complex and enhances their transcriptional activity.^[^ ^20a^ ^]^ Taken together, these data suggest that Tcf3 and Tcf4 bind to the *Il9* promoter and block positive regulators BATF, IRF4 and Smad3 but not PU.1, and that Id1 effectively prevents Tcf3 and Tcf4 from performing this activity.

Next, we examined whether differentiation of Th9 cells is affected when Tcf3 or Tcf4 in WT cells is downregulated by shRNA‐mediated KD. There was no change in Th9 cell differentiation upon KD of either Tcf3 or Tcf4 (Figure 4G–J). In addition, KD of Tcf12 did not affect expression of IL‐9 (Figure S4A,B). One possibility for this result is that when the amount of Id1 is sufficiently high, Tcf3 and Tcf4 cannot bind to the *Il9* promoter region, regardless of their expression levels. In this case, reduction of Tcf3 and Tcf4 in WT T cells should not affect differentiation of Th9 cells. To test this possibility, we performed KD of Tcf3 or Tcf4 in *Id1*‐deficient CD4 T cells. However, Tcf3 or Tcf4 single gene KD did not affect Th9 cell differentiation (Figure 4K). This suggests a degree of complexity with respect to the nature of TCF proteins, and that other members of the family may compensate for their activity. Interestingly, we found that *Tcf4* levels increased in *Id1*‐deficient Th9 cells transduced with the control shRNA vector (Figure S4C, Supporting Information). To confirm this, we measured *Tcf3* and *Tcf4* levels in *Id1*‐deficient Th9 cells and found that only *Tcf4* levels increased (Figure S4D, Supporting Information), suggesting that Id1 also regulates expression of Tcf4.

### 2.5 Id1‐Deficient Th9 Cells Show Increased Expression of Genes Related to Type 1 Immune Responses

**Figure 5:**
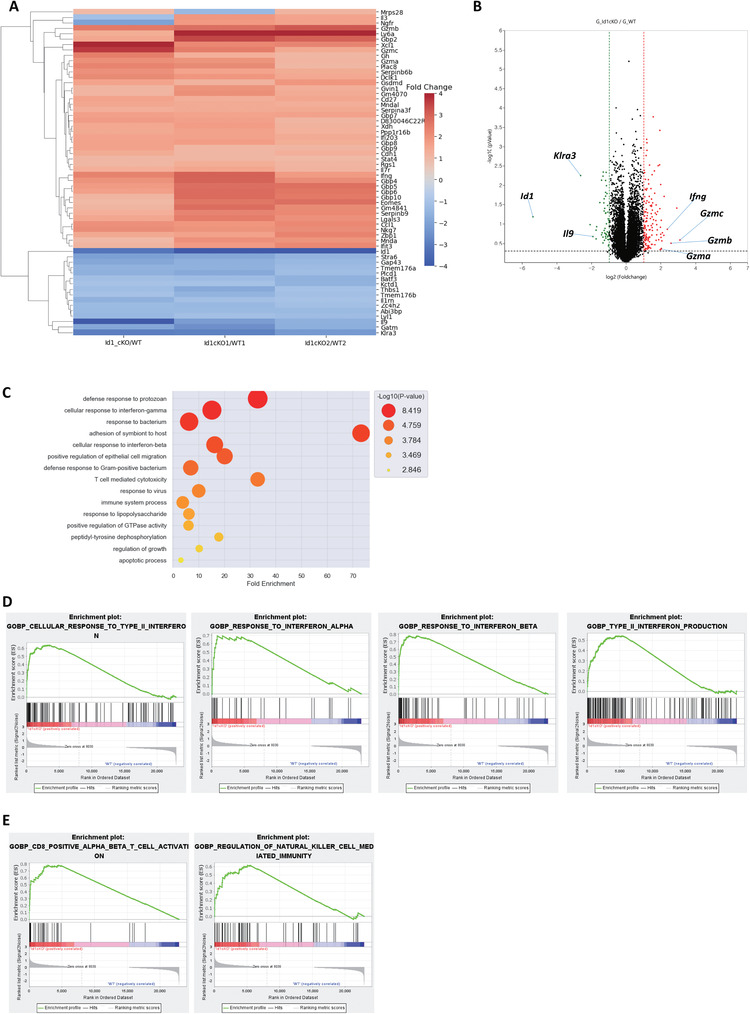
Global gene expression analyses. A) RNA‐seq was performed using RNA isolated from WT or *Id1* cKO Th9 cells. Clustering heat map derived from RNA‐seq data. B) Volcano plot of RNA‐seq data. C) DAVID graphic results for the RNA‐seq data. Gene categories enriched in KO were shown. D,E) Gene set enrichment analysis (GSEA).

To gain insight into the molecular mechanisms underlying Id1‐mediated Th9 cell differentiation, we analyzed global gene expression profiles in WT and *Id1* cKO Th9 cells by RNA‐sequencing (RNA‐seq). Consistent with the results above, expression of *Il9* by *Id1*‐deficient Th9 cells fell markedly (**Figure** **5**A,B), but that of known transcription factor genes important for Th9 cell differentiation (i.e., *Irf4*, *Spi1*, *Batf*, *Stat6*, and *Gata3*) did not change (Data S2, Supporting Information). Interestingly, expression of genes associated with Th1 cells and cytotoxic T cells (i.e., *Cd8*, *Gzma*, *Gzmb*, *Gzmc*, and *Ifng)* increased in *Id1*‐deficient Th9 cells (Figure 5A,B). Next, we examined changes in biological processes in *Id1* cKO Th9 cells using the DAVID gene ontology analysis (https://david.ncifcrf.gov/summary.jsp). Overall, processes of “cellular response to interferon gamma and beta” were increased in *Id1* cKO Th9 cells (Figure 5C). Moreover, gene set enrichment analysis (GSEA) revealed that gene sets related to “cellular response to type I (IFN‐α, IFN‐β) and type II (IFN‐γ) interferon”, “type II interferon production”, “CD8 T cell activation”, “natural killer cell mediated immunity” were upregulated in *Id1* cKO Th9 cells (Figure 5D,E), further supporting the results of gene ontology analysis. Type I and II interferons have a role in supporting the type I immune response, which triggers the activation of Th1, CD8 T, and natural killer cells to combat viral infections. These results suggest that *Id1* deficiency causes the characteristics of Th9 cells to change from type II to type I.

### 2.6 Id1‐Deficient Th9 Cells Ameliorate Airway Inflammation in an Animal Model of Asthma

**Figure 6:**
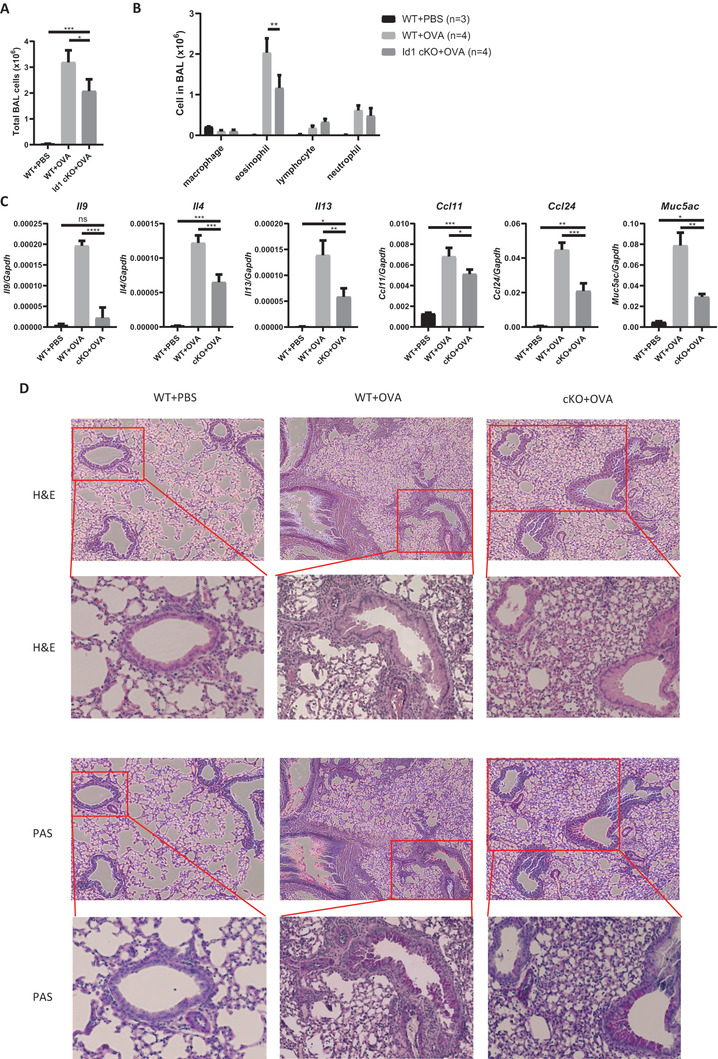
*Id1* cKO mice show ameliorated airway inflammation. A) Total cells were isolated from lung BAL fluids and counted using a hemocytometer; *n* = 3–4. B) Isolated BAL cells were stained with Diff Quik to identify different cell types and then counted. C) mRNA was isolated from lung tissue, and expression levels were measured by RT‐qPCR. D) Lung tissue was fixed in 4% formaldehyde and stained with H&E or PAS before histological analysis. Original magnification, × 100 and × 200. E) dLN cells were isolated and stimulated in the presence of OVA for 24 h. CD4 T cells were sorted and then *Id1* and *Il9* mRNA expression was measured by RT‐qPCR. Error bars represent the standard deviation. *p*‐values were analyzed by A–C) one‐way ANOVA/Tukey's test or E) by Student's *t*‐test. ^*^ *p* < 0.05, ^**^ *p* < 0.01, ^***^ *p* < 0.001, ^****^ *p* < 0.0001.

Finally, to investigate the functional alterations in *Id1‐*deficient CD4 T cells in vivo, we used an animal model of asthma. Airway inflammation in WT or *Id1* cKO mice was induced by sensitization and challenge with ovalbumin (OVA). Total cell numbers in the bronchoalveolar lavage (BAL) fluid of *Id1* cKO mice were lower than those in WT mice (**Figure** **6**A). Next, we analyzed cells in the BAL fluid from these mice. Although the number of macrophages, lymphocytes, and neutrophils in WT and Id1 cKO mice was comparable, the number of eosinophils was lower in the latter (Figure 6B). Next, we isolated lung tissues from the mice and measured mRNA expression. Expression of *Il9* mRNA in *Id1* cKO mice was much lower than that in WT mice (Figure 6C). Expression of *Il4* and *Il13* mRNA was also reduced in *Id1* cKO mice, although less markedly than *Il9* (Figure 6C). In addition, *Ccl11*, *Ccl24*, and *Muc5ac*, which correlate with asthma,^[^ ^28^ ^]^ decreased in *Id1* cKO mice (Figure 6C). Inflammatory cell infiltration and mucus production by lung tissue, as observed by or hematoxylin and eosin (H&E) and periodic acid Schiff (PAS) staining, respectively, were less pronounced in *Id1* cKO mice than in WT mice (Figure 6D). To assess *Id1* expression in this setting, we isolated draining lymph node cells from the OVA‐immunized mice, stimulated them in the presence of OVA for 24 h, and sorted CD4 T cells from the ex vivo cultured cells. The expression of *Id1* as well as *Il9* was significantly increased in CD4 T cells from the asthma‐induced mice (Figure 6E), highlighting the physiological significance of Id1.

**Figure 7:**
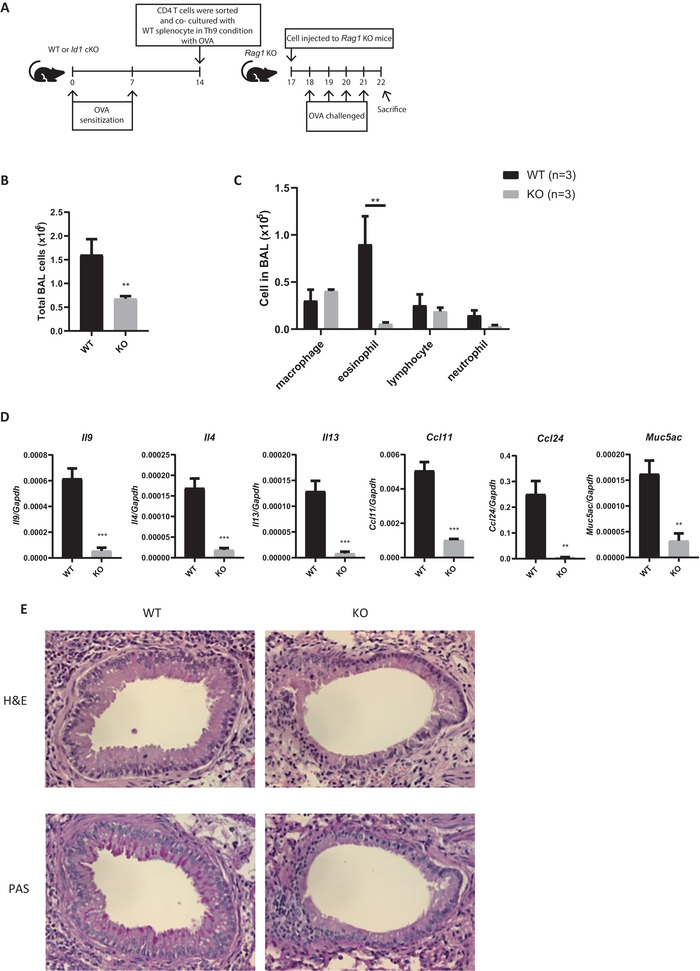
*Id1‐*deficient Th9 cells ameliorate airway inflammation in a cell‐intrinsic manner. A) Schematic diagram showing the adoptive transfer model of airway inflammation. B) Total BAL cells were isolated from the lung and counted using a hemocytometer; *n* = 3. C) Isolated BAL cells were stained with Diff Quik to identify different cell types and then counted. D) mRNA was isolated from lung tissue, and expression levels were measured by RT‐qPCR. E) Lung tissue was fixed in 4% formaldehyde and stained with H&E or PAS before histological analysis. Original magnification, × 400. Error bars represent the standard deviation. *p*‐values were determined by Student's *t*‐test. ^**^ *p* < 0.01, ^***^ *p* < 0.001.

Next, to determine whether *Id1‐*deficient Th9 cells reduce airway inflammation in a cell‐intrinsic manner, we used an adoptive transfer model (**Figure** **7**A). CD4 T cells were isolated from OVA‐challenged WT or *Id1* cKO mice and stimulated under Th9‐polarizing conditions (Figure S5A, Supporting Information). The cells were then adoptively transferred into *Rag1*‐deficient mice, which were then challenged with OVA. Recipients of WT Th9 cells developed airway inflammation; however, this inflammation was ameliorated in recipients of *Id1* cKO Th9 cells (Figure 7). The total cell number in BAL fluid and the number of eosinophils were lower in *Id1* cKO Th9 cell‐transferred mice than in WT Th9 cell‐transferred mice (Figure 7B,C). To assess the stability and proliferation capacity of the adoptively transferred CD4 T cells, we isolated CD4 T cells from the lungs of the recipient mice and measured expression of *Il9* and surface expression of Ki‐67. The pattern of *Il9* expression in both WT and *Id1* cKO CD4 T cells was preserved before and after transfer (Figure S5A,B, Supporting Information), and the proliferation capacity of the transferred cells remained comparable between WT and *Id1* cKO CD4 T cells after transfer (Figure S5B, Supporting Information). These findings suggest that the transferred cells retained their functional characteristics. Moreover, in line with the reduction in BAL eosinophil numbers (Figure 7C), the percentage of lung eosinophils was also reduced (Figure S5C, Supporting Information). Furthermore, expression of genes whose expression correlates with asthma, including *Il9*, *Il4*, *Il13*, *Ccl11*, *Ccl24*, and *Muc5ac*, was much lower in recipients of *Id1* cKO Th9 cells (Figure 7D). Inflammatory cell infiltration and mucus production in the lung were also less pronounced in recipients of *Id1* cKO Th9 cells (Figure 7E). Taken together, these data indicate that *Id1*‐deficient Th9 cells ameliorate airway inflammation in a cell‐intrinsic manner.

## 3 Discussion

In this study, we examined the function of Id1 during Th9 cell differentiation. *Id1*‐deficient naïve CD4 T cells harbored a specific defect with respect to differentiation into Th9 cells. Studies of *Id1* heterozygote, Id1 KD, and Id1 GE cells revealed that IL‐9 expression was dependent on *Id1* levels. Id1 interacted with Tcf3 and Tcf4. Tcf3 and Tcf4 inhibited expression of IL‐9 in Th9 cells by binding to the *Il9* promoter region. Id1 effectively inhibited this activity of Tcf3 and Tcf4. *Id1*‐deficient CD4 T cells ameliorated airway inflammation in an animal model of asthma. Collectively, these data suggest that Id1 acts as an essential positive regulator of Th9 cell differentiation by inhibiting the transcriptional activity of Tcf3 and Tcf4.

Our study shows that *Id1* is essential for Th9 cell differentiation. CD4 T cells from both *Id1* cKO mice and whole *Id1* KO mice did not differentiate into Th9 cells. In addition, Id1 suppressed differentiation into Th9 cells in a dose‐dependent manner, as shown in the experiments using *Id1* heterozygous cells or cells transduced with *Id1*‐shRNA or *Id1*‐sgRNA. Moreover, Th9 cell differentiation was upregulated by overexpression of Id1. The dosage effect suggests that relative ratio of Id1 and its binding partners is important in regulating the partners’ activity. Because Id1 dominantly inhibits its partners, partial reduction of Id1 will cause changes in the ratio, and thus partners’ activity.

Although Id1 binding partners Tcf3 and Tcf4 act as negative regulators of Th9 cells, they did not affect expression of other transcription factors, including BATF, IRF4, PU.1, STAT6, and GATA3. Instead, they bound to the *Il9* promoter region and inhibited expression of *Il9*. Thus, it seems that they block the transcription factors’ transcription‐enhancing activity. Tcf3 and Tcf4 play essential roles in T cell development and homeostasis^[^ ^29^ ^]^; however, they also inhibit Th9 cell differentiation. Paradoxically, IL‐4 and TGF‐β signals upregulate expression of Tcf3 and Tcf4 in Th9 cells. Therefore, their activity in Th9 cells should be restrained by Id1. We propose that Th9‐specific expression of Id1 ensures proper differentiation of Th9 cells.

It seems that both Tcf3 and Tcf4 are important for blockade of Th9 cell differentiation. Single gene knockdown of *Tcf3* or *Tcf4* in *Id1* cKO cells could not restore IL‐9 levels. This suggests that even if one gene is inhibited, other members of the family may block Th9 cell differentiation. In addition, since expression of *Tcf4*, but not *Tcf3*, is increased in *Id1*‐deficient Th9 cells, *Tcf4* expression seems to be regulated by Id1. Thus, there are multiple ways in which Id1 inhibits Tcf3 and Tcf4.

Interestingly, *Id1*‐deficient Th9 cells showed increased expression of genes related to type I immune responses. RNA‐seq data revealed increases in expression of *Ifng*, *Gzma*, *Gzmb*, *Gzmc*, and *Cd8*. DAVID gene ontology and GSEA analyses revealed upregulation of the gene categories “cellular response to type I (IFN‐α, IFN‐β) and type II (IFN‐γ) interferon”, “type II interferon production”, “CD8 T cell activation”, “natural killer cell‐mediated immunity” in *Id1*‐deficient Th9 cells. Th9 cells mediate type II immune responses by activating mast cells and eosinophils, and are associated with allergic reactions and asthma.^[^ ^30^ ^]^ By contrast, type I immunity stimulates macrophages to produce IFN‐γ, which activates NK cells and CD8 T cells to kill cells infected with viruses.^[^ ^30^ ^]^ We speculate that *Id1* deficiency reduces IL‐9 production, which causes the characteristics of Th9 cells to change from type II to type I. One possible reason for this change is that IL‐9 antagonizes type I immune responses since each CD4 T cell subset cross regulates each other.

Th9 cells express not only IL‐9 but also Granzyme B. However, we found that in *Id1*‐deficient Th9 cells, *Il9* was completely depleted, whereas *Gzmb* was highly upregulated. Granzyme B is regulated by Eomes.^[^ ^31^ ^]^ Consistent with this, we found that *Eomes* was upregulated in *Id1*‐deficient Th9 cells. The Granzyme family in Th9 cells is regulated by the PU.1‐Traf6‐Eomes cascade^[^ ^32^ ^]^; however, our RNA‐seq data revealed no changes in expression of *Spi1* (encoding PU.1) or *Traf6* in *Id1*‐deficient Th9 cells. Thus, it seems that there are other ways by which the Granzyme family is regulated, and that Id1 acts as a negative regulator of the process. Based on our data, we suggest that *Id1* not only regulates expression of *Il9* but also plays a role in maintaining the functional characteristics of Th9 cells.

We found that *Id3*, as well as *Id1*, positively regulates Th9 cell differentiation. Contrary to our study, another group reported that *Id3* acts as a negative regulator.^[^ ^15^ ^]^ *Id1* and *Id3* belong to the same family of proteins. *Id3* can form a heterodimer with E‐protein^[^ ^33^ ^]^ and induces thymocyte development by blocking E‐protein [34]. Although both Id1 and Id3 act as positive regulators, Id1 seems to be the more powerful factor in Th9 cell differentiation because it is more efficient at blocking Tcf3 or Tcf4, and because overexpression of Id1 led to a greater increase in *Il9*.

In summary, we found that Id1 is an essential positive regulator of Th9 cell differentiation by inhibiting Tcf3 and Tcf4. The detailed underlying mechanisms and possible therapeutic implications of targeting Id1 warrant further study.

## 4 Experimental Section

### Mice

Female C57BL/6J mice (6–10 weeks old) were purchased from Daehan Bio Link. *Cd4^cre^*, *Rag1*‐deficient, and Gt(ROSA)26Sor^tm1.1(CAG‐cas9*,‐EGFP)Fezh^ (Cas9 KI) mice were purchased from Jackson Laboratory. *Id1^fl/fl^* mice were generously provided by Dr. Jonathan Keller, National Cancer Institute–Frederick, and *Id1^−/−^* (whole *Id1* KO) mice were generously provided by Dr. Robert Benezra, Sloan Kettering Institute. *Id1^fl/fl^* mice were crossed with *Cd4^cre^* mice to obtain *Id1^fl/+^‐Cd4^cre^* (*Id1* hetero) mice and *Id1^fl/fl^‐Cd4^cre^* (*Id1* cKO) mice. All mice were cared for under specific pathogen‐free conditions. All animal experiments were approved by the Sogang University Institutional Animal Care and Use committee (approval no. IACUCSGU2019_09).

### Isolation and Differentiation of CD4 T Cells

Naïve CD4 T cells were isolated from mouse spleens using the MojoSort Ms CD4 naïve T cell Isolation Kit (BioLegend, cat #: 480 040). The isolation step is described in a previous study.^[^ ^18^ ^]^ Naïve CD4 T cells were cultured in RPMI 1640 (Gibco, cat #: 22400‐089) supplemented with 10% fetal bovine serum (FBS, SERANA, cat #: S‐FBS‐US‐015), MEM amino acids (Gibco, cat #: 11140‐051), non‐MEM amino acids (Gibco, cat #: 11140‐050), penicillin‐streptomycin (Gibco, cat #: 15140‐122), and 2‐mercaptoethanol (Gibco, cat #: 21985‐023). Plate‐bound anti‐CD3 (10 µg mL^−1^; 2C11) and soluble anti‐CD28 (2 µg mL^−1^) antibodies were used in all cultures. Th0 cells were induced by incubation with IL‐2 (1 ng mL^−1^, R&D, cat #: 402‐ML) for 3 days. Th1 cells were induced by incubation with an anti‐IL‐4 antibody (5 µg mL^−1^; 11B11), mouse recombinant IL‐2 (1 ng mL^−1^), and mouse recombinant IL‐12 (3.5 ng mL^−1^, eBioscience, cat #: 14‐8121‐62). Th2 cells were induced by incubation with an anti‐IFN‐γ antibody (5 µg mL^−1^; XMG1.2), mouse recombinant IL‐2 (1 ng mL^−1^), and mouse recombinant IL‐4 (20 ng mL^−1^, R&D, cat #: 404‐ML). Th9 cells were induced by incubation with an anti‐IFN‐γ antibody (5 µg mL^−1^), mouse recombinant IL‐4 (40 ng mL^−1^), and human recombinant TGF‐β1 (2 ng mL^−1^, R&D, cat #: 240‐B). Th17 cells were induced by incubation with an anti‐IL‐4 antibody (10 µg mL^−1^), an anti‐IFN‐γ antibody (5 µg mL^−1^), mouse recombinant IL‐6 (50 ng mL^−1^, R&D, cat #: 406‐ML), human recombinant TGF‐β1 (2 ng mL^−1^), mouse recombinant IL‐1β (2 ng mL^−1^, eBioscience, cat #: PMC0814), and mouse recombinant TNF‐α (1 ng mL^−1^, eBioscience, cat #: 14‐8321‐63). Treg cells were induced by incubation with anti‐IL‐4 (5 µg mL^−1^) and anti‐IFN‐γ antibodies (5 µg mL^−1^), mouse recombinant IL‐2 (0.4 ng mL^−1^), and human recombinant TGF‐β1 (5 ng mL^−1^). All subsets were cultured for 3 days.

### Flow Cytometry

To analyze T cell differentiation, cells were stimulated for 4 h with PMA (50 ng mL^−1^, Sigma, cat #: P8139), ionomycin (1 µm, Sigma, cat #: I0634), and Brefeldin A (BioLegend, cat #: 420 601). Next, cells were harvested and fixed in BD Cytofix/Cytoperm buffer (cat #: 51–2090KZ) or FOXP3 Fix/Perm buffer (BioLegend, cat #: 421 401), followed by addition of BD Perm/Wash (cat #: 51–2090KZ) or FOXP3 Perm Buffer (BioLegend, cat #: 421 s402). The cells were then stained with a PE‐conjugated anti‐IL‐9 antibody (BioLegend, cat #: 514 103), a PE‐conjugated anti‐IFN‐γ antibody (BioLegend, cat #: 505 807), a PE‐conjugated anti‐IL‐13 antibody (BioLegend, cat #: 159 403), a PE‐conjugated anti‐IL‐17 antibody (BioLegend, cat #: 506 904), an APC‐conjugated anti‐IL‐9 antibody (BioLegend, cat #: 514 105), and an APC‐conjugated anti‐Foxp3 antibody (eBioscience, cat #: 17‐5773‐82). To analyze apoptosis of cells transduced with overexpression vectors, cells were stained with a PE‐conjugated anti‐Annexin V (BioLegend, cat #: 640 908) antibody and 7‐AAD Viability Staining Solution (BioLegend, cat #: 420 403). To analyze lymphocyte development in the thymus of *Id1* cKO mice, cells were isolated from the thymus and then stained with an APC‐conjugated anti‐CD3ε antibody (BioLegend, cat #: 100 235), a FITC‐conjugated anti‐CD4 antibody (BioLegend, cat #: 100 509), and a PE‐conjugated anti‐CD8 antibody (BioLegend, cat #: 100 707). Stained cells were analyzed using a FACSCalibur or an Accuri C6 plus cytometer (BD Biosciences).

### ELISA

Naïve CD4 T cells were cultured under Th9‐polarizing conditions for 3 days. Then, the cells were restimulated by PMA and Ionomycin for 4 h. The amount of IL‐9 in the supernatants was measured by IL‐9 Mouse Uncoated ELISA Kit (Thermo Fisher, cat #: 88‐8092‐88)

### RNA Isolation and Reverse Transcription Quantitative Polymerase Chain Reaction (RT‐qPCR)

RNA was isolated from cultured cells using TRIZol reagent (Molecular Research Center, Inc. cat #: TR118), and cDNA was synthesized by reverse transcription (RT) using TOPscript RT (Enzynomics, cat #: RT002L). Real‐time quantitative polymerase chain reaction (qPCR) was performed using TOPreal qPCR 2X PreMIX SYBR green (Enzynomics, cat #: RT500M), or TOPreal qPCR 2X PreMIX Taqman probe (Enzynomics, cat #: RT600M), and a Roche LightCycler 96. The primer sequences are listed in Table S1 (Supporting Information).

### Retroviral Transduction

On day 0, 1.2 × 10^6^ Phoenix Eco cells were cultured in a plate. On day 1, the Phoenix Eco cells were transfected with the MIEG3, shRNA, or sgRNA vector (2 µg), along with the pcl‐Eco helper vector (1 µg). On day 2, the Phoenix Eco cell medium was replaced with fresh medium, and naïve CD4 T cells were cultured under Th0 conditions. On day 3, the T cell medium was replaced with virus‐containing medium (i.e., supernatants from Phoenix Eco cells) supplemented with polybrene (5 µg mL^−1^, Sigma, cat #: TR‐1003) for infection by spin down (1600 × g for 90 min at 25 °C). After spin down, the virus‐containing medium was replaced with Th1, 2, 9, 17, or Treg medium and cultured for an additional 2 days. Transduced cells were analyzed to detect GFP signals.

### Co‐IP Analysis

Co‐IP and immunoblot analyses were performed as described.^[^ ^18^ ^]^ Protein A/G PLUS‐Agarose (Santa Cruz, cat #: sc‐2003) and the following antibodies were used for Co‐IP: anti‐FLAG (Sigma, cat #: F1804), anti‐Id1 (Santa Cruz, cat #: sc‐13310), and normal mouse anti‐IgG (Santa Cruz, cat #: sc‐2025). Anti‐FLAG (Sigma), anti‐Id1 (Santa Cruz), and HRP‐conjugated anti‐mouse IgG antibodies were used for immunoblot analysis.

### ChIP

Differentiated Th9 cells (5 × 10^6^ cells) transduced with MIEG3‐empty, MIEG3‐*Flag‐Tcf3‐*, or MIEG3‐*Flag‐Tcf4‐*expressing vectors were harvested and fixed with 1% formaldehyde. The ChIP‐assay was performed using a Magna ChIP A/G kit (Millipore, cat #: Magna0017). For immunoprecipitation, cells were incubated overnight at 4 °C with an anti‐FLAG antibody (Sigma). qPCR was performed with precipitated DNA and the primers listed in Table S2 (Supporting Information).

### RNA‐seq

WT and *Id1* cKO naïve CD4 T cells were differentiated into Th9 cells. The cells were then dissolved in TRIZol reagent, and RNA was isolated. The amount of RNA was measured in an ND 2000 spectrophotometer (Thermo Fisher, Waltham, MA, USA). For the control and experimental RNAs, libraries were constructed using the QuantSeq 3 mRNA Seq Library Prep Kit (Lexogen, Inc., Austria). High throughput sequencing was performed (single‐end 75 sequencing) using the NextSeq 500 platform (Illumina, Inc., USA). Data were analyzed by the ExDEGA program (eBiogen Inc).

### Mass Spectrometry

Naïve CD4 T cells were stimulated and transduced with MIEG3‐empty or MIEG3‐*Id1‐* expressing vectors and cultured under Th9‐polarizing conditions. Cells were harvested, dissolved in IP 150 buffer, and sonicated to obtain lysates. Cell lysates were precleared by incubation with protein A/G (Santa Cruz) and then incubated overnight with normal IgG (Santa Cruz) or an anti‐Id1 antibody (Santa Cruz). Antibody‐treated samples were precipitated for 2 h with protein A/G and then incubated with SDS loading dye prior to separation in SDS‐PAGE gels. The gel containing precipitated proteins was sent to BASIL Biotech (Korea) for mass spectrometry.

### Transient Reporter Assay

A Dual‐Luciferase Reporter Assay System (Promega, cat #: E1910) was used for the transient reporter assay. On day 0, EL4 cells (2.0 × 10^6^) were transfected with a pGL3 luciferase reporter vector (10 µg), a pCMV protein expression vector (10 µg), and a pRL *Renilla* luciferase control vector (100 ng) by electroporation. On day 1, the cells were stimulated for 4 h with PMA (50 ng mL^−1^) and ionomycin (1 µm). Enzyme activity was measured in a LB96V luminometer (Berthold), and *Firefly* luciferase activity was normalized to that of *Renilla* luciferase.

### Animal Model of Asthma

On days 0 and 7, WT mice were injected intraperitoneally with PBS or OVA (20 µg, Sigma, cat #: A5503) mixed with aluminum hydroxide gel. *Id1* cKO mice were injected with OVA (20 µg, Sigma) mixed with aluminum hydroxide gel. On day 14, mice were aerosol‐challenged for 40 min with 1% OVA in PBS; this was repeated on 4 consecutive days. On day 18, mice were sacrificed for analysis.

### Adoptive Transfer Model of Asthma

On day 0 and 7, WT and *Id1* cKO mice were injected intraperitoneally with OVA (20 µg) mixed with aluminum hydroxide gel. On day 14, CD4 T cells were isolated from the spleen. To obtain antigen‐presenting cells, splenocytes were isolated from WT mice, and CD4 T cells were removed by negative selection using magnetic beads. CD4 T cells (1 × 10^6^) and splenocytes (1 × 10^7^) were cocultured for 3 days in Th9‐culture medium supplemented with OVA (200 µg mL^−1^). On day 17, the cells were transferred into *Rag1*‐deficient mice. On days 18–21, recipient mice were aerosol‐challenged for 40 min with 1% OVA in PBS. On day 22, mice were sacrificed for analysis. Cells were sorted using a FACSAriaIII cytometer (BD Biosciences).

### Analysis of Asthma‐Induced Mice

BAL fluid was isolated from the lung using PBS and a syringe. After centrifugation, cells were harvested for counting and Diff Quik (Sysmex, cat #: 38 721) staining. Morphology and staining characteristics were analyzed.

Lungs were harvested from mice and used for cell analysis or staining or RNA isolation.

For cell analysis, the lungs were digested with collagenase A (1 mg mL^−1^, Roche, cat #: COLLA‐RO) and DNase I (0.5 µg mL^−1^, Sigma, cat #: DN25) in HBSS for 30 min. To isolate cells, the lungs were minced on a cell strainer and red blood cells were lysed using ACK Lysing buffer (Gibco, cat #: A1049201). The cells were then harvested and stimulated with PMA, ionomycin and Brefeldin A for 4 h in RPMI1640 medium supplemented with 10% fetal bovine serum. After stimulation, the cells were fixed and stained for analysis by flow cytometry. For staining, the lungs were fixed in 4% formaldehyde solution. After 24 h, lung tissue was stained with H&E or PAS. For RNA isolation, lung tissue was homogenized in TRIZol reagent, RNA was isolated, and cDNA was synthesized prior to RT‐qPCR.

### Statistical Analysis

Data are expressed as the mean ± standard deviation (SD). Differences between two groups were analyzed using Student's *t* test. Difference among more than two groups was analyzed by one‐way ANOVA, followed by Tukey's post hoc test. *p*‐values <0.05 were considered statistically significant (^*^ *p* < 0.05, ^**^ *p* < 0.01, and ^***^ *p* < 0.001, ^****^ *p* < 0.0001).

## Conflict of Interest

The authors declare no conflict of interest.

## Supporting information

Supporting Information

Supplemental Dataset 1

Supplemental Dataset 2

## Data Availability

The data that support the findings of this study are available in the supplementary material of this article.
