# Supplementary material for: Transcription Factor Id1 Plays an Essential Role in Th9 Cell Differentiation by Inhibiting Tcf3 and Tcf4
Source: Adv Sci (Weinh). 2023 Oct 22;10(35):2305527. doi: 10.1002/advs.202305527 (PMC10724384; doi:10.1002/advs.202305527)
Supplement: Supplementary file 1 — Supporting Information [file ADVS-10-2305527-s003.pdf]

ADVANCED SCIENCE Open Access

Supporting Information

for *Adv. Sci.*, DOI 10.1002/advs.202305527

Transcription Factor Id1 Plays an Essential Role in Th9 Cell Differentiation by Inhibiting Tcf3 and Tcf4

*Woo Ho Lee, Kyung Jin Hong, Hua-Bing Li and Gap Ryol Lee**

Supplementary figures and tables

Fig. S1. Naïve CD4 T cells from whole *Id1* KO mice cannot differentiate into Th9 cells.

Fig. S2. Th9 cell differentiation is dependent on the level of *Id1* expression.

Fig. S3. Tcf12 does not blocks Il9 promoter activity, and Tcf3 and Tcf4 do not block PU.1-IRF4 activity on Il9 promoter.

Fig. S4. Id1 regulates *Tcf4* expression.

Fig. S5. Cell analysis of adoptive transfer model by flow cytometry.

Table S1. Primer sequences for RT-qPCR

Table S2. Primer sequences for Chip-PCR

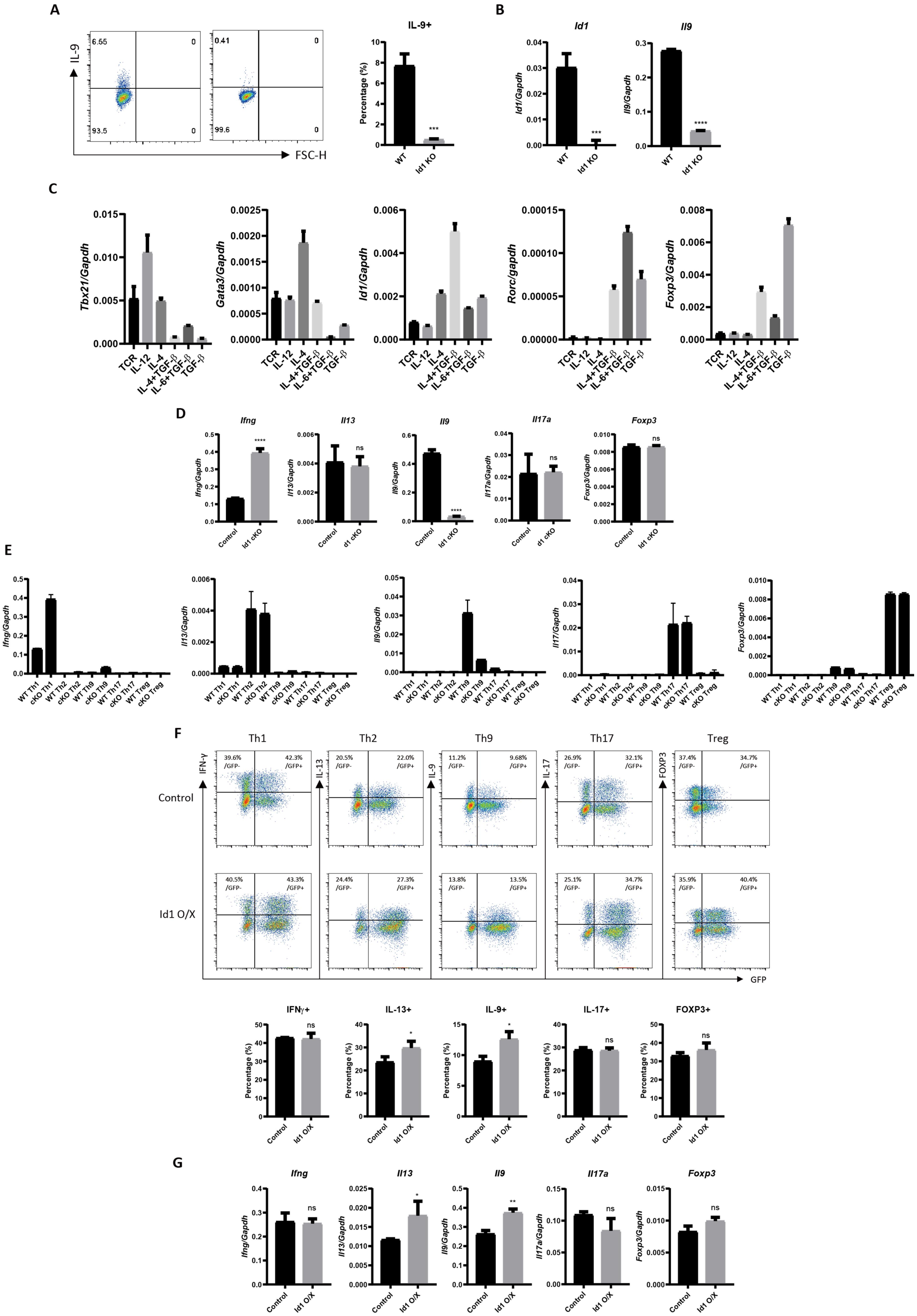
A
IL-9
FSC-H
6.55
0
93.5
0
0.41
0
99.6
0
IL-9+
Percentage (%)
WT
Id1 KO
***
B
Id1
Id1/Gapdh
WT
Id1 KO
***
Il9
Il9/Gapdh
WT
Id1 KO
****
C
Tbx21/Gapdh
Gata3/Gapdh
Id1/Gapdh
Rorc/gapdh
Foxp3/Gapdh
TCR
IL-12
IL-4
IL-4+TGF-β
IL-6+TGF-β
TGF-β
D
Ifng
Ifng/Gapdh
Control
Id1 cKO
****
Il13
Il13/Gapdh
Control
d1 cKO
ns
Il9
Il9/Gapdh
Control
Id1 cKO
****
Il17a
Il17a/Gapdh
Control
d1 cKO
ns
Foxp3
Foxp3/Gapdh
Control
Id1 cKO
ns
E
Ifng/Gapdh
Il13/Gapdh
Il9/Gapdh
Il17/Gapdh
Foxp3/Gapdh
WT Th1
cKO Th1
WT Th2
cKO Th2
WT Th9
cKO Th9
WT Th17
cKO Th17
WT Treg
cKO Treg
F
Th1
Th2
Th9
Th17
Treg
Control
Id1 O/X
IFN-γ
IL-13
IL-9
IL-17
FOXP3
GFP
39.6% /GFP-
42.3% /GFP+
20.5% /GFP-
22.0% /GFP+
11.2% /GFP-
9.68% /GFP+
26.9% /GFP-
32.1% /GFP+
37.4% /GFP-
34.7% /GFP+
40.5% /GFP-
43.3% /GFP+
24.4% /GFP-
27.3% /GFP+
13.8% /GFP-
13.5% /GFP+
25.1% /GFP-
34.7% /GFP+
35.9% /GFP-
40.4% /GFP+
IFNγ+
IL-13+
IL-9+
IL-17+
FOXP3+
Percentage (%)
Control
Id1 O/X
ns
*
*
ns
ns
G
Ifng
Il13
Il9
Il17a
Foxp3
Ifng/Gapdh
Il13/Gapdh
Il9/Gapdh
Il17a/Gapdh
Foxp3/Gapdh
Control
Id1 O/X
ns
*
**
ns
ns

Figure S1. Naïve CD4 T cells from whole *Id1* KO mice cannot differentiate into Th9 cells. Naïve CD4 T cells were isolated from the spleen of WT or whole *Id1* KO mice and cultured for 3 days under Th9-polarizing conditions. **(A and B)** IL-9 was measured by flow cytometry, and mRNA levels were measured by RT-qPCR. **(C)** Naïve CD4 T cells were stimulated for 24 hours with TCR in the presence of the indicated cytokines. Key transcription factor of each subset was measured by RT-qPCR. **(D)** mRNA level of each subset was measured by RT-qPCR. **(E)** Expression of subset-specific marker genes in each subset was measured by RT-qPCR. **(F and G)** *Id1* was overexpressed in each subset. The frequency of cytokine expressing cells was measured by flow cytometry, and mRNA levels were measured by RT-qPCR. **(A and F)** Flow cytometry and **(B, C, D, E, G)** RT-qPCR data are representative of three independent experiments. The data in the bar graph next to the flow cytometry data were pooled from three independent experiments. The error bars represent the standard deviation. P-values were determined by Student's *t*-test. ***$P < 0.001$, ****$P < 0.0001$.

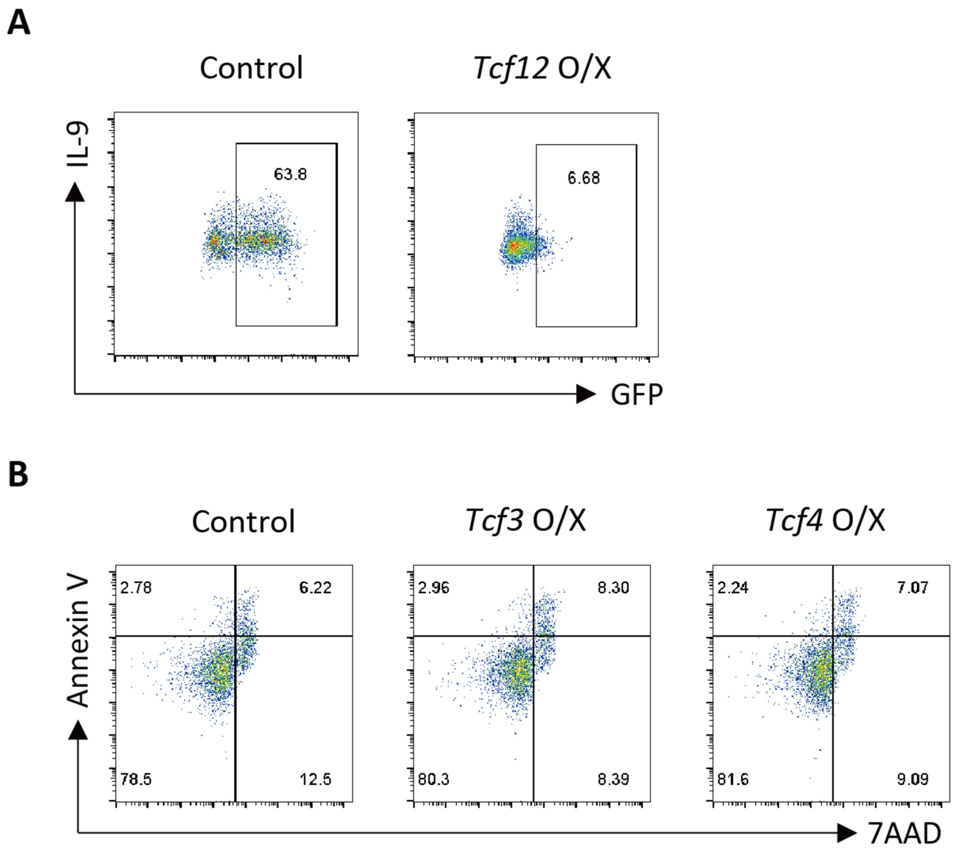

Figure S2. The effect of overexpression of *Tcf3*, *Tcf4*, *and Tcf12* on cell survival, and regulation of *Tcf3* and *Tcf4* by cytokines. Naïve CD4 T cells were cultured under Th0 conditions for 1 day. The cells were then transduced with *Tcf3*, *Tcf4*, or *Tcf12* expression vectors and cultured under Th9-polarizing conditions for 2 days. Tcf3- and Tcf4-overexpressing cells were stained with Annexin V and 7AAD to measure cell viability.

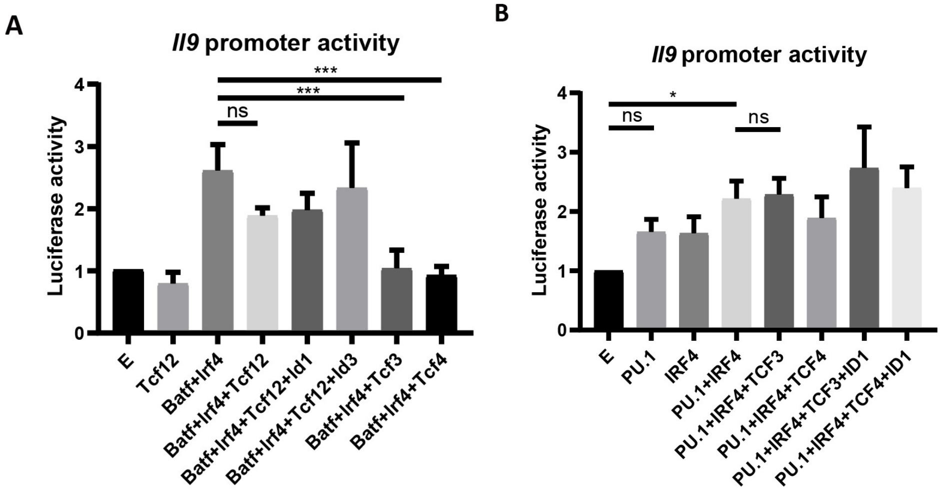

Figure S3. Tcf12 does not blocks *Il9* promoter activity and Tcf3, Tcf4 do not block PU.1-IRF4 activity on *Il9* promoter. On day 0, EL4 cells were transduced with vectors containing various transcription factors, along with the *Il9* promoter. The cells were then stimulated with PMA and ionomycin for 4 hours, and promoter activity was measured on day 1. **(A)** The effect of Tcf12 on *Il9* promoter in the presence of BATF+IRF4. **(B)** The effect on Tcf3 or Tcf4 effect on *Il9* promoter in the presence of PU.1+IRF4. Data were pooled from three independent experiments. The error bars represent the standard deviation. P-values were determined by one-way ANOVA/Tukey's test. ns: not significant, ***P < 0.001.

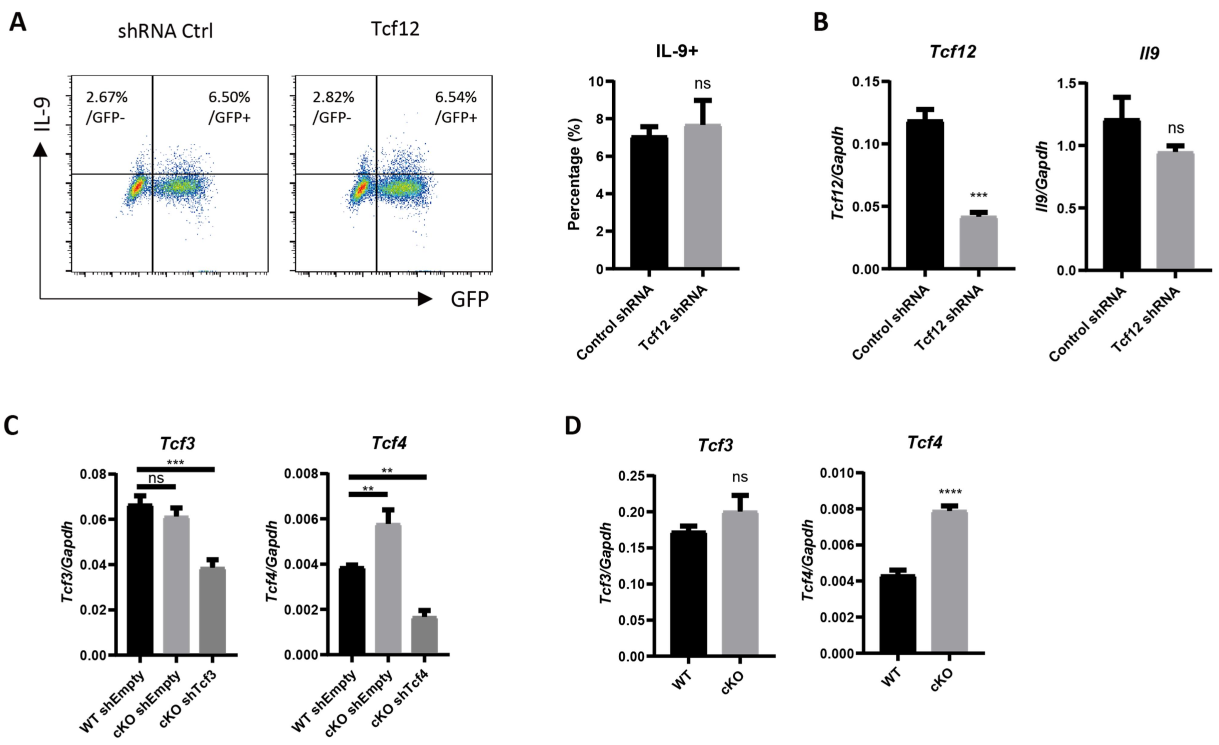

Figure S4. Id1 regulates *Tcf4* expression. **(A, B)** Naïve CD4 T cells were transduced with a *Tcf12*-shRNA vector and cultured under Th9-polarizing conditions. mRNA levels were measured by RT-qPCR. **(C)** *Tcf3* and *Tcf4* mRNA levels in **Figure 4J** were measured by RT-qPCR. **(D)** *Tcf3* and *Tcf4* mRNA levels in WT or *Id1* cKO Th9 cells were measured by RT-qPCR. **(A)** Flow cytometry and **(B, C, D)** RT-qPCR data are representative of three independent experiments. The data in the bar graph next to the flow cytometry data were pooled from three independent experiments. The error bars represent the standard deviation. **(A, B, D)** P-values were determined by Student's *t*-test. **(C)** P-values were determined by one-way ANOVA/Tukey's test. ns: not significant, $^{**}P < 0.01$, $^{***}P < 0.001$, $^{****}P < 0.0001$.

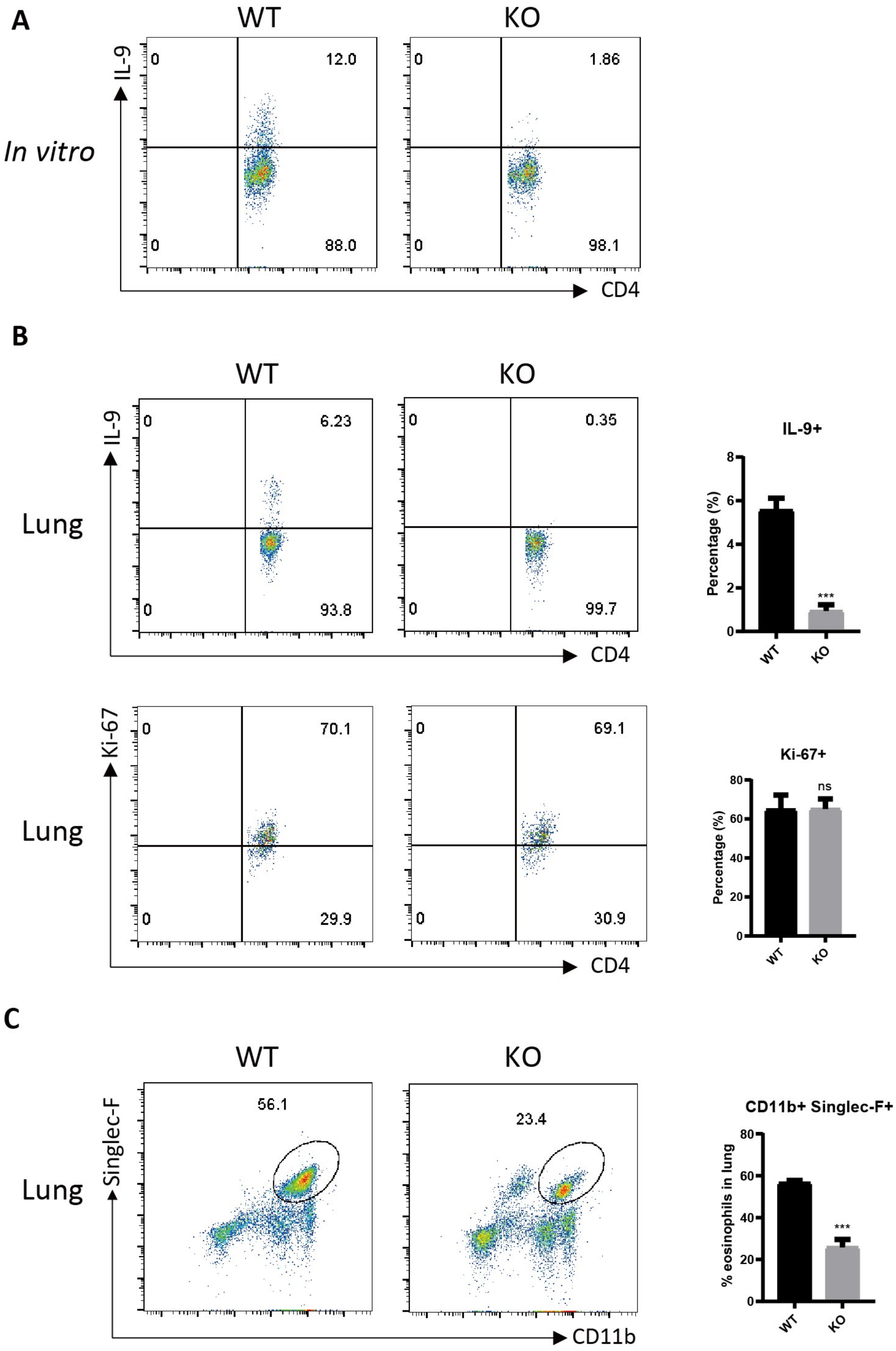
A
WT
KO
IL-9
In vitro
0
12.0
0
88.0
0
1.86
0
98.1
CD4
B
WT
KO
IL-9
Lung
0
6.23
0
93.8
0
0.35
0
99.7
CD4
IL-9+
Percentage (%)
***
WT
KO
Ki-67
Lung
0
70.1
0
29.9
0
69.1
0
30.9
CD4
Ki-67+
Percentage (%)
ns
WT
KO
C
WT
KO
Singlec-F
Lung
56.1
23.4
CD11b
CD11b+ Singlec-F+
% eosinophils in lung
***
WT
KO

Figure S5. Analysis of adoptive transferred cells by flow cytometry (n = 3). **(A)** Cell preparation for adoptive transfer. CD4 T cells were FACS-sorted from the sensitized mice and cultured with WT splenocyte and OVA under Th9-polarizing conditions for 3 days. **(B)** CD4 T cells were isolated from the lung of the recipient mice after adoptive transfer and analyzed by flow cytometry. **(C)** CD45+ cells were gated and then Singlec-F+ CD11b+ cells were analyzed by flow cytometry. **(B and C)** The data in the bar graph next to the flow cytometry data were pooled from 3 mice in each group. The error bars represent the standard deviation. **(B and C)** P-values were determined by Student's t-test. ***P < 0.001

Table S1. RT-qPCR primers

Gapdh Fw (5’-3’) CAATGTGTCCGTCGTGGATCT

Gapdh Rv (5’-3’) GTCCTCAGTGTAGCCCAAGATG

Gapdh Probe CGTGCCGCCTGGAGAAACCTGCC

Ifng Fw (5’-3’) GGATGCATTCATGAGTATTGC

Ifng Rv (5’-3’) CCTTTTCCGCTTCCTGAGG

Ifng Probe TTTGAGGTCAACAACCCACAGGTCCA

Il13 Fw (5’-3’) GCTTATTGAGGAGCTGAGCAACA

Il13 Rv (5’-3’) GGCCAGGTCCACACTCCATA

Il13 Probe CAAGACCAGACTCCCCTCTGCAACG

Il17a Fw (5’-3’) CTCCAGAAGGCCCTCAGACTAC

Il17a Rv (5’-3’) AGCTTTCCCTCCGCATTGACACAG

Il17a Probe TCTGGGAAGCTCAGTGCCGCCACCAGC

Foxp3 Fw (5’-3’) CCCAGGAAAGACAGCAACCTT

Foxp3 Rv (5’-3’) TTCTCACAACCAGGCCACTTG

Foxp3 Probe ATCCTACCCACTGCTGGCAAATGGAGTC

Il9 Fw (5’-3’) ACCACACCGTGCTACAGGGA

Il9 Rv (5’-3’) TCGCAGGAAAAGGACGGACACG

Id1 Fw (5’-3’) TGG ACG AGC AGC AGG TGA ACG

Id1 Rv (5’-3’) GCA CTG ATC TCG CCG TTC AGG

Id3 Fw (5’-3’) CTT GGA CGA CAT GAA CCA CT

Id3 Rv (5’-3’) AGG TCG AGG ATG TAG TCT ATG

Tcf3 Fw (5’-3’) CGC ACC AGC AGT ACA GAT GAG

Tcf3 Rv (5’-3’) CAG CTT GGT CTG CGC CTT A

Tcf4 Fw (5’-3’) GCC TCT TCA CAG TAG TGC CAT

Tcf4 Rv (5’-3’) TCC CTG TTG TAG TCG GCA GT

Tcf12 Fw (5’-3’) ATG TGC TAC GAA ACC ATG CAG

Tcf12 Rv (5’-3’) GCC ATT GAG ACT GAC TGA ATC TT

Ccl11 Fw (5’-3’) GAATCACCAACAACAGATGCAC

Ccl11 Rv (5’-3’) ATCCTGGACCCACTTCTTCTT

Ccl24 Fw (5’-3’) ATTCTGTGACCATCCCCTCAT

Ccl24 Rv (5’-3’) TGTATGTGCCTCTGAACCCAC

Muc5ac Fw (5’-3’) GTGGTTTGACACTGACTTCCC

Muc5ac Rv (5’-3’) CTCCTCTCGGTGACAGAGTCT

Table S2. ChIP-qPCR primers

Il9 Ebox site 1 Fw (5’-3’) TGCTCTTCAGTTCTGTGCTG

Il9 Ebox site 1 Rv (5’-3’) CCCAGTCCCCTAAATCTACC

Il9 Ebox site 2 Fw (5’-3’) CAGAGATGCAGCACCACAT

Il9 Ebox site 2 Rv (5’-3’) ATCCTCCAGCAGATGACTC

Il9 CNS0 Fw (5’-3’) ATGCGGAATGGGTTTTCACT

Il9 CNS0 Rv (5’-3’) AAGCTCCACACACTTAGTTTGT

Il9 CNS1 Fw (5’-3’) CCCTGTAACTCACTGTCTATCAGC

Il9 CNS1 Rv (5’-3’) GCAGGAATTCTGGTTGTGAG

Il9 CNS1a Fw (5’-3’) CAGTCTACCAGCATCTTCCAGTCTAGC

Il9 CNS1a Rv (5’-3’) GTGGGCACTGGGTATCAGTTTGATGTC

Il9 CNS2 Fw (5’-3’) TCACCCACTTTAGTCCTTTCAAAA

Il9 CNS2 Rv (5’-3’) AATTACAGAATTTTGCCCCAGGTCCTG
